# *NTRK1*-related Hereditary Sensory and Autonomic Neuropathy Type 4: The Role of the Histamine Challenge Test

**DOI:** 10.1177/2329048X221108826

**Published:** 2022-06-20

**Authors:** Amytice Mirchi, Julie Richer, Maryam Oskoui, Hugh J. McMillan

**Affiliations:** 1 Department of Pediatrics, Neurology and Neurosurgery, 5620 McGill University, Montreal, Canada; 2 Department of Medical Genetics, Children’s Hospital of Eastern Ontario, Ottawa, Canada

**Keywords:** NTRK1 protein, human, hereditary sensory and autonomic neuropathy type 4, congenital insensitivity to pain with anhidrosis, histamine phosphate, intradermal tests, small fiber neuropathy

## Abstract

Hereditary sensory and autonomic neuropathies (HSAN) are rare, genetically inherited disorders characterized by impaired unmyelinated nerve fiber function. Here we report a patient with self-mutilation behavior and decreased response to pain, suggestive of an underlying small fiber neuropathy. Nerve conduction studies were normal but sympathetic skin response was absent at the left arm. Intradermal histamine challenge test was performed to evaluate the function of small unmyelinated nerve fibers and revealed absence of a flare response. Using whole genome sequencing, a novel variant in the neurotrophic tyrosine kinase type 1 gene was identified, expanding the known disease-causing variants associated with HSAN type 4. Through this case, we demonstrate the role of the histamine challenge test in patients suspected to have a small fiber neuropathy where electrophysiological testing may be normal and who may present with non-specific symptoms including hypotonia and failure to thrive. The information gained can guide genetic testing and contribute to interpretation of new variants identified.

## Introduction

Hereditary sensory and autonomic neuropathies (HSAN) are a group of disorders that share the common feature of impaired or absent function of small unmyelinated nerves causing decreased or absent pain sensation. HSAN are otherwise heterogeneous with each type showing variability in age of onset, mode of inheritance as well as variable involvement of larger myelinated nerve fibers and variable autonomic features (Table 1).^1–3^

**Table 1.** Associated Genes, Clinical Features, Electrophysiological and Nerve Pathology in HSAN Types 1 to 5.

	HSAN type 1	HSAN type 2	HSAN type 3	HSAN type 4	HSAN type 5
Inheritance	AD	AR	AR	AR	AR
Gene(s)	*SPTLC1, SPLTC2, ATL1, RAB7* ^a^ *DNMT1*	*WNK1, FAM134B, KIF1A*	*IKBKAP*	*NTRK1*	*NGFB*
Age of onset	Second to fifth decade	Birth	Birth	Birth	Birth
Sensory symptoms					
Pain perception	Absent distally	Absent	Mild to moderate decrease	Absent	Absent
Self-mutilation	Rare	Frequent	Rare	Frequent	Frequent
Temperature perception	Absent distally	Significantly reduced to absent	Mild to moderate decrease	Absent	Normal
Vibration sense	Normal	Normal	Normal	Normal to moderate decrease	Normal
Deep tendon reflexes	Normal to decreased	Frequently decreased	Decrease	Normal to decreased	Normal
Autonomic symptoms					
Postural hypotension	Absent	Uncommon	Frequent	Uncommon	Absent
Temperature instability	Absent	Absent	Common	Common^b^	Absent
Episodic hypertension	Absent	Rare	Frequent	Rare	Absent
Histamine challenge test	Abnormal	Abnormal	Abnormal	Abnormal	Abnormal
Skin features	Ulcerations, thickened and callused	Ulcerations, thickened and callused, erythematous blotching	Erythematous blotching	Dry, hyperkeratotic, lichenified ulcerations, dystrophic nails, hypotrichosis	Ulcerations
Fractures / Charcot joints	Common with advancing age	Uncommon	Common	Frequent	Frequent
Scoliosis	Uncommon	Common	Frequent	Uncommon	Absent
Cognitive impairment	May be present (mild)	Common	Uncommon	Common	Absent
Nerve conduction study	Low normal to mildly reduced motor and sensory velocities and action potential (late finding)	Highly abnormal sensory nerve conduction with absent potential; low normal motor nerve conduction	Normal to mildly reduced motor and sensory velocities and action potential, second CMAP may be seen	Normal to mildly reduced motor and sensory velocities and action potential	Normal to mildly reduced motor and sensory velocities and action potential
Nerve biopsy findings	Distal loss of unmyelinated and small myelinated fibers, mild decrease in large myelinated fibers	Loss of large and small myelinated fibers, mild decrease in number of unmyelinated fibers	Loss of unmyelinated and small myelinated fibers, loss of autonomic neurons, mild decrease in large myelinated fibers	Absence of unmyelinated fibers, decreased small myelinated fibers, loss of sudomotor fibers	Loss of unmyelinated and small myelinated fibers

Legend: Rare <1%; uncommon <30%; common 30–65%; frequent >65%. AD, autosomal dominant; AR, autosomal recessive, CMAP, compound motor action potential; IKBKAP, Inhibitor of kappa light polypeptide gene enhancer in B-cells, kinase complex-associated protein, ^a^*RAB7* variants also linked to Charcot-Marie-Tooth disease type 2B (CMT2B), ^b^At risk of hyperpyrexia secondary to anhidrosis. Table expanded from Axelrod F et al (2007).^2^

HSAN type 4 (HSAN4), also known as congenital insensitivity to pain with anhidrosis, is characterized by profound insensitivity to pain, impaired temperature sensation and anhidrosis.^1,4^ Biallelic pathogenic variants in neurotrophic tyrosine kinase type 1 (*NTRK1*) (MIM: 191315) have been shown to cause HSAN4 through a loss of function mechanism.^1,4^

We report a girl who initially presented with hypotonia and global developmental delay. At 13-month of age, she developed self-mutilation injuries. The histamine challenge testing was key to identifying impaired function of small unmyelinated nerves. Although the histamine challenge test has been used for decades^5^ it remains a valuable functional tool that can help direct genetic testing as well as to help interpret previously unreported variants.

## Case

A 21-month-old girl first presented to medical attention at 11 months old. She had mild axial hypotonia and global developmental delay as well as increased feeding difficulties and failure to thrive. She had been born at term after an uneventful pregnancy and delivery. Her birth weight was 3.21 kg (45^th^ percentile). Her weight gain progressively slowed; at 8 months and 10 months old she weighted 7.6 kg (30^th^ percentile and 13^th^ percentile, respectively) and at 12 months old she weighed 7.2 kg (third percentile). Her head circumference at 11 months was 43.5 cm (15^th^ percentile). She had a history of intermittent fevers that occurred with or without symptoms of infection. Her parents were third cousins and of eastern African ancestry. Parents and two older sisters were healthy. On examination at 11 months of age, she was alert and visually attentive. Her muscle strength was normal but she showed axial and appendicular hypotonia with hyporeflexia (1 + ) to biceps patella and ankle. The remainder of her examination was within normal limits. Chromosomal microarray (aCGH) and genetic testing for Prader-Willi syndrome, myotonic dystrophy and spinal muscular atrophy were negative. MRI brain was unrevealing. A nasogastric tube was placed and she showed steady weight gain.

**Figure 1.**
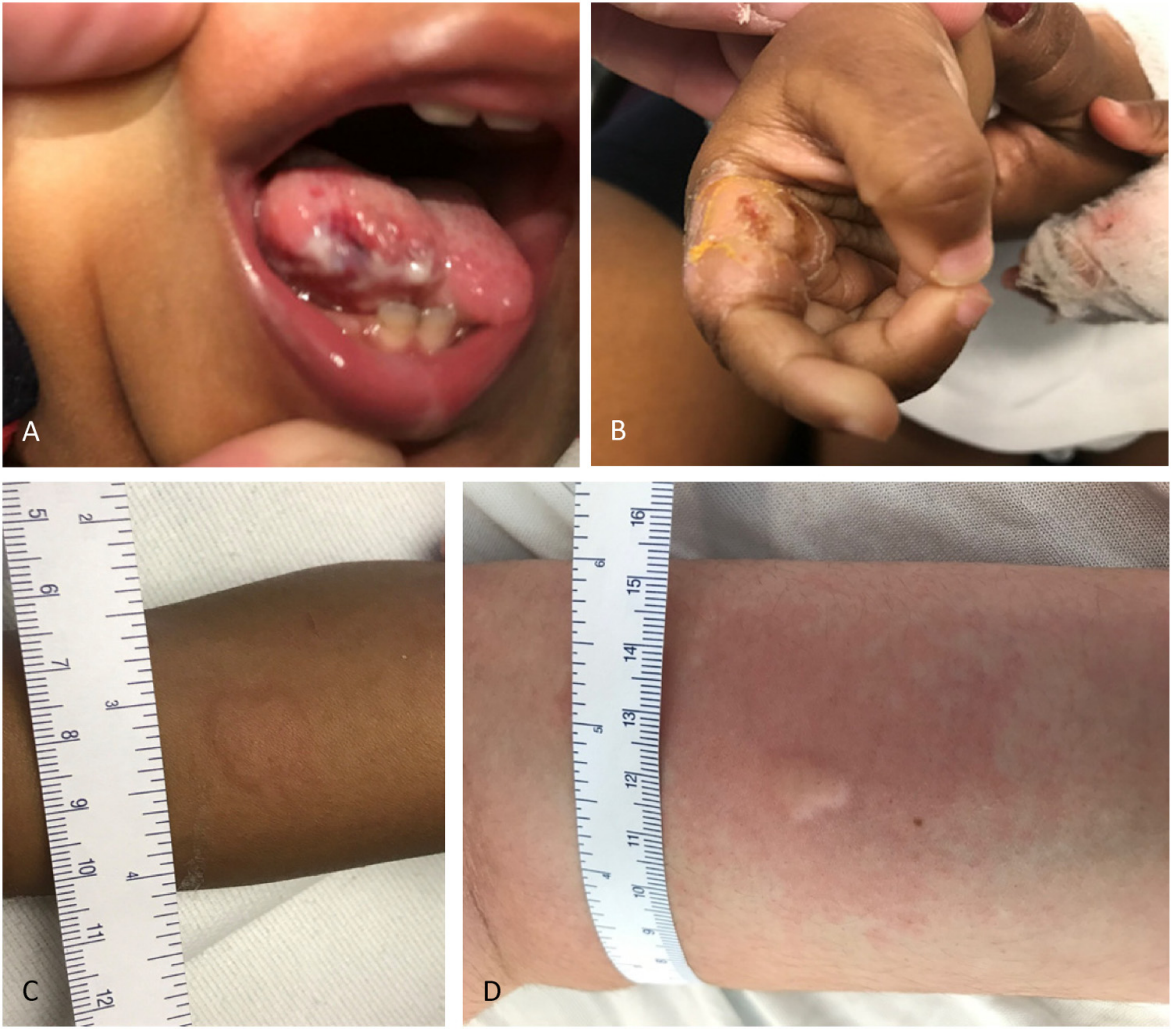
Patient at 21 months old showing self-mutilation injury to tongue (A) and fingers (B). Histamine challenge testing of the patient revealed central area of pallor (1.5 cm^2^) but a complete absence of surrounding erythema (C) confirming functional absence of small autonomic fiber function. Normal control (D) shows an area of erythema extending several centimeters beyond the central area of pallor.

At 13 months old she developed self-mutilation injuries first involving her tongue. Subsequently, she had bit a portion of her tongue and had multiple bites on her fingers and hands with deep bites to the region of her first dorsal interosseus bilaterally at various stages of healing (Figure 1A and B). In retrospect, her parents recalled absence of pain with phlebotomy however she had cried when a needle had been used to place an intravenous line in a scalp vein. When crying she did produce tears. Although parents had reported her to have fever during infancy, they noted that she did not sweat like their older children. Developmentally she was sitting but not cruising or walking independently. She had only two spoken words. Her neurological examination revealed normal cranial nerves and normal strength. She did have a mildly high arched palate, axial and appendicular hypotonia and absent reflexes in her lower extremities. Nerve conduction studies revealed an absent sympathetic skin response to her left upper arm but normal median, radial and sural sensory responses as well as normal median nerve motor response (to abductor pollicis brevis) and tibial nerve motor response (to abductor hallucis). Needle electromyography was not performed. Given the clinical suspicion for a small fiber neuropathy, an intradermal histamine challenge test was performed.^2,5^ The histamine challenge test protocol involves the intradermal administration of histamine phosphate 0.1 mL (0.275 mg/mL) instilled in her ventral forearm^2,5^ She demonstrated no response to the administration of histamine phosphate, specifically a central area of pallor or wheal at the site of injection with no surrounding erythema (Figure 1C).^2,5^ For comparison a control subject shows a normal response as indicated be the sizeable area of erythema (Figure 1D). Given her multisystem involvement, whole genome sequencing was obtained at 15 months of age which revealed a homozygous variant in *NTRK1* (c.2153 G > A; p.Trp718*) inherited in-trans from each parent. This variant was predicted to be likely pathogenic by the American College of Medical Geneticsguidelines given its absence from healthy controls (PM2) and is predicted to result in a premature stop codon leading to truncated or absent protein (PVS1).^6^ This prediction, in addition to the clinical phenotype and evidence of functional abnormality of small unmyelinated nerve fiber allowed for a definite clinical diagnosis to be made.

## Discussion and Conclusion

HSAN4 is part of a group of rare disorders known as the hereditary sensory and autonomic neuropathies (HSAN). This group of heterogenous diseases affect the small unmyelinated and in some types, myelinated nerves giving rise to decreased or absent pain and temperature sensation as well as variable autonomic dysfunction.^1,2,7^ The *NTRK1* gene located on chromosome 1q23.1 encodes the receptor tyrosine kinase type 1 which binds to nerve growth factor.^1,7^ Downstream signaling allows the development and survival of nociceptive sensory and sympathetic neurons.^1,7^ As illustrated by our clinical report, self-mutilation behavior most often involving the lip and digits is a frequent phenotypic characteristic of HSAN4 as children begin to teethe and explore their surroundings.^1,4,7^ Patients with HSAN4 are also at increased risk of corneal scarring and painless bone fractures in addition to intellectual disability and emotional lability.^1,4,7^ The profound absence of unmyelinated neurons and sensory insensitivity present in patients with HSAN4 result in high frequency of self-mutilation behaviors that can help distinguish it from other HSANs (Table 1). Preserved lacrimation and fungiform papillae on the tongue helps to differentiate HSAN4 from HSAN type 3. The development of anhidrosis and associated cutaneous changes are additional important distinguishing features.^1,2^

Similarly to our patient, nerve conduction studies may be normal in patients with HSANs given that these primarily evaluate the large myelinated fibers, highlighting the importance of ancillary testing when evaluating a patient suspected to have a small fiber peripheral neuropathy. As shown through the diagnostic evaluation of our patient, we believe that the histamine challenge test has an important role in the evaluation of these patients. This test is based on the absence of a flare response in HSAN as it relies on antidromic conduction of the unmyelinated c-fibers.^2^ Limitations of the histamine challenge test should however be recognized. This includes the possibility of false-negative tests in young infants particularly below 6 months of age who show decreased skin reactivity to histamine, patients taking a medication with anti-histaminic properties and false-negative results due to technical factors including sub-optimal injection of the histamine-containing solution and interval time between the injection and evaluation for a flare response being too short or too long.^8,9^

Other tests that can be considered in the evaluation of a sensory and/or autonomic small fiber peripheral neuropathy include pathological studies consisting of a peripheral nerve biopsy. Typically, the sural sensory nerve will be biopsied and ultrastructual examination used to evaluate the relative populations of myelinated and unmyelinated fibers. Intraepidermal nerve fiber density is another technique consisting of a skin biopsy using a skin punch biopsy tool followed by small nerve fibers quantification within the epidermis and the sweat glands.^10^

In summary, our clinical report contributes to the expansion of identified disease-causing variants in *NTRK1* leading to HSAN4. Our patient's diagnostic journey demonstrates the importance of pursuing ancillary testing despite normal nerve conduction studies when suspecting a small fiber peripheral neuropathy. More particularly, the histamine challenge test is simple, reliable and will demonstrate an absent flare response in patients with aberrant nociceptive nerve fibers.^2,5^

## Abbreviations

CMT: Charcot-Marie-Tooth disease
HSAN: Hereditary sensory and autonomic neuropathy
HSAN4: Hereditary sensory and autonomic neuropathy type 4
aCGH: Chromosomal microarray
EMG: Electromyography
NTRK1: Neurotrophic tyrosine kinase type 1
ACMG: American College of Medical Genetics
